# Sirtuin 3 Downregulation in *Mycobacterium tuberculosis*-Infected Macrophages Reprograms Mitochondrial Metabolism and Promotes Cell Death

**DOI:** 10.1128/mBio.03140-20

**Published:** 2021-02-02

**Authors:** Lorissa J. Smulan, Nuria Martinez, Michael C. Kiritsy, Chido Kativhu, Kelly Cavallo, Christopher M. Sassetti, Amit Singhal, Heinz G. Remold, Hardy Kornfeld

**Affiliations:** a Department of Medicine, University of Massachusetts Medical School, Worcester, Massachusetts, USA; b Department of Microbiology and Physiological Systems, University of Massachusetts Medical School, Worcester, Massachusetts, USA; c Infectious Disease Horizontal Technology Centre, Agency for Science, Technology and Research (A*STAR), Singapore, Singapore; d Singapore Immunology Network, Agency for Science, Technology, and Research (A*STAR), Singapore, Singapore; e Department of Medicine, Brigham and Women’s Hospital, Harvard Medical School, Boston, Massachusetts, USA; Harvard School of Public Health

**Keywords:** *Mycobacterium tuberculosis*, cell death, macrophages, mitochondrial metabolism, sirtuin

## Abstract

Mycobacterium tuberculosis induces metabolic reprogramming in macrophages like the Warburg effect. This enhances antimicrobial performance at the expense of increased inflammation, which may promote a pathogen-permissive host environment. Since the NAD^+^-dependent protein deacetylase Sirtuin 3 (SIRT3) is an important regulator of mitochondrial metabolism and cellular redox homeostasis, we hypothesized that SIRT3 modulation mediates M. tuberculosis-induced metabolic reprogramming. Infection of immortalized and primary murine macrophages resulted in reduced levels of SIRT3 mRNA and protein and perturbation of SIRT3-regulated enzymes in the tricarboxylic acid cycle, electron transport chain, and glycolytic pathway. These changes were associated with increased reactive oxygen species and reduced antioxidant scavenging, thereby triggering mitochondrial stress and macrophage cell death. Relevance to tuberculosis disease *in vivo* was indicated by greater bacterial burden and immune pathology in M. tuberculosis-infected *Sirt3*^−/−^ mice. CD11b^+^ lung leukocytes isolated from infected *Sirt3^−/−^* mice showed decreased levels of enzymes involved in central mitochondrial metabolic pathways, along with increased reactive oxygen species. Bacterial burden was also greater in lungs of *LysM^cre^Sirt3^L2/L2^* mice, demonstrating the importance of macrophage-specific SIRT3 after infection. These results support the model of SIRT3 as a major upstream regulatory factor, leading to metabolic reprogramming in macrophages by M. tuberculosis.

## Abstract (precis)

Tuberculosis, the disease caused by the bacterium M. tuberculosis, remains one of the top 10 causes of death worldwide. Macrophages, the first cells to encounter M. tuberculosis and critical for defense against infection, are hijacked by M. tuberculosis as a protected growth niche. M. tuberculosis-infected macrophages undergo metabolic reprogramming where key mitochondrial pathways are modulated, but the mechanisms driving this metabolic shift is unknown.

## INTRODUCTION

Macrophages are the first immune cells to encounter inhaled Mycobacterium tuberculosis. The bacillus blocks phagosome maturation, reprograms metabolism, and induces cell death in infected macrophages (1). Infection by M. tuberculosis skews macrophage metabolism from oxidative phosphorylation (OXPHOS) toward aerobic glycolysis, comparable to the Warburg effect. This altered metabolic state may represent a host defense to fuel a rapid inflammatory response (2–6). However, Hackett et al. (7) reported repression of glycolysis following persistent M. tuberculosis infection in macrophages, which depended on upregulation of microRNA-21. Cumming et al. (8) described decelerated flux through both glycolysis and OXPHOS in human macrophages following infection. Despite these contrasting studies, available evidence consistently points toward mitochondrial metabolic reprogramming initiated by the bacillus. Mitochondria are a major source for reactive oxygen species (ROS) in macrophages and participate in M. tuberculosis-activated apoptotic and necrotic cell death (9, 10), raising the question whether shared mechanisms underlie perturbation of macrophage metabolism and cell fate.

Cellular metabolism is controlled by energy sensors that include sirtuins (SIRTs) (11, 12), a family of seven NAD^+^-dependent protein deacetylases with distinct subcellular localizations and target proteins (13). SIRT3, the major mitochondrial sirtuin, regulates central metabolism, energy balance, and cellular redox (14–18) and is a regulator of metabolic reprogramming in cancer cells (19). Kim et al. recently reported that SIRT3 enhances anti-M. tuberculosis activity in macrophages by mediating autophagy, preserving mitochondrial function, and limiting ROS accumulation (20). However, the role SIRT3 plays in the metabolic reprogramming and ROS-mediated death of infected macrophages remains unexplored.

Here, we show that M. tuberculosis infection of macrophages downregulated SIRT3 in a TLR2-dependent manner, resulting in perturbation of enzymes in the tricarboxylic acid (TCA) cycle, electron transport chain (ETC), and glycolytic pathways. This led to increased mitochondrial ROS (mtROS), and cell death. Using SIRT3-deficient primary bone-marrow derived macrophages (BMDM) and CD11b^+^ lung leukocytes isolated from M. tuberculosis-infected *Sirt3^−/−^* mice, we show that the changes in TCA, ETC, and glycolytic enzymes were regulated *in vitro* and *in vivo* in a SIRT3-dependent manner. Finally, SIRT3 was shown to be required for optimal host protection against M. tuberculosis infection *in vivo*. We propose that SIRT3 modulation by M. tuberculosis is a key upstream event leading to metabolic reprogramming and macrophage dysfunction in tuberculosis (TB).

## RESULTS

### Altered metabolic profile in *M. tuberculosis*-infected macrophages.

**FIG 1:**
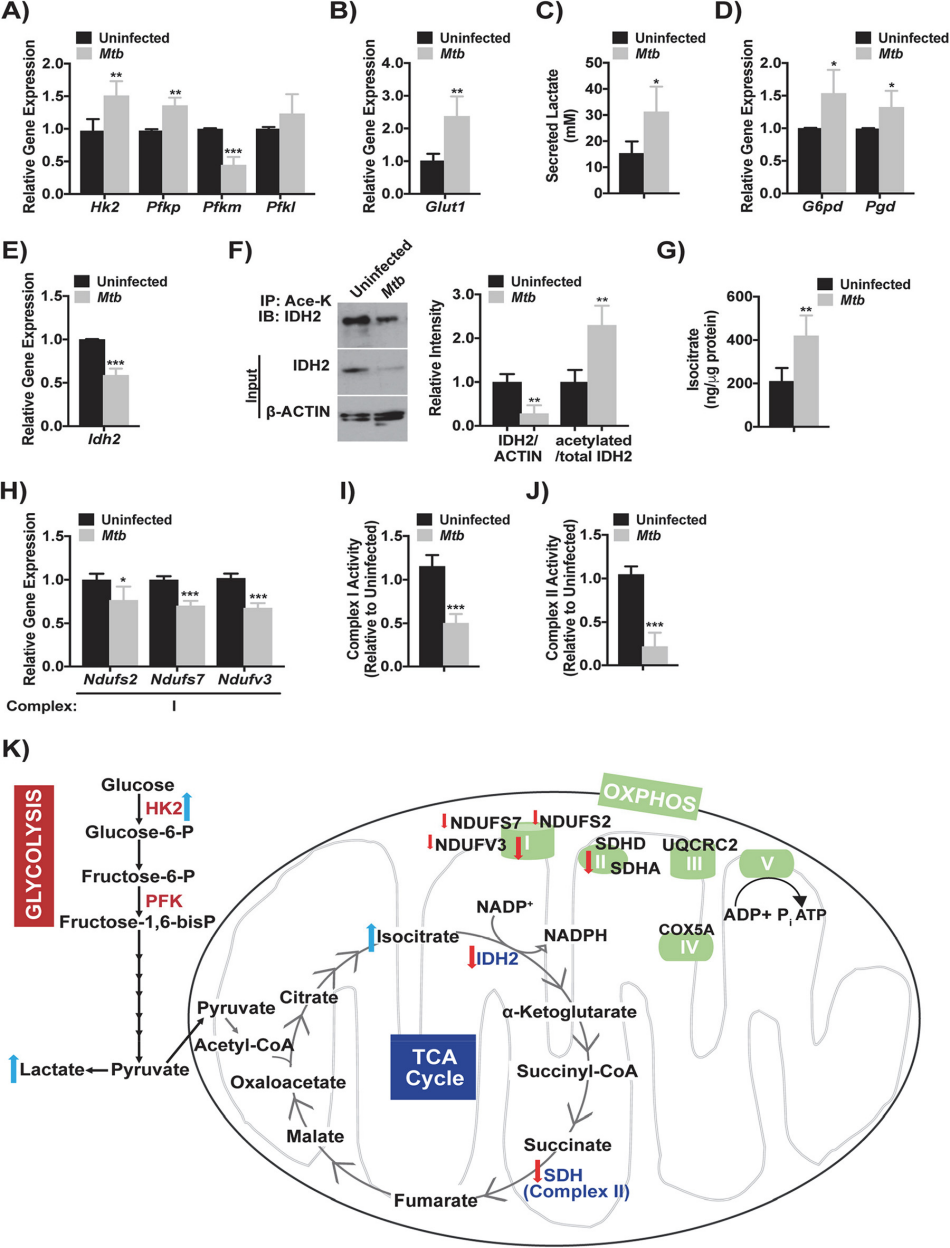
Altered metabolic profile in M. tuberculosis-infected macrophages. (A and B) Gene expression of hexokinase-2 (*Hk2*) and phosphofructokinase, platelet (*Pfkp*), muscle (*Pfkm*), and liver (*Pfkl*) type (A) and glucose transporter 1 (*Glut1*) (B) in J2 macrophages at 24 h p.i. with M. tuberculosis (MOI of 10). The data are expressed relative to uninfected macrophages and show the means ± the SD of four independent experiments. (C) Secreted lactate levels from J2 macrophages with identical conditions as in panel A. Error bars show the means ± the SD of four independent experiments. (D and E) Gene expression of glucose-6-phosphate dehydrogenase (*G6pd*) and 6-phosphogluconate dehydrogenase (*Pgd*) (D) and isocitrate dehydrogenase 2 (*Idh2*) (E) in J2 macrophages with identical conditions and analysis as in panel A. (F, left) Representative immunoblot showing total IDH2 or β-Actin and acetylated IDH2 in J2 macrophage cell lysate following immunoprecipitation with acetyl-lysine with identical infection conditions as in panel A. (Right) Densitometry of immunoblots showing total IDH2 normalized to β-actin and total to acetylated IDH2. The data are expressed relative to uninfected macrophages and show the means ± the SD of four independent experiments. (G) Isocitrate levels in J2 macrophage total cell lysate infected as in panel A. Error bars show the means ± the SD of four independent experiments. (H) Gene expression of subunits from complex I of the ETC in J2 macrophages with identical infection conditions and analysis as in panel A. *Ndufs2* and *Ndufs7*, NADH:ubiquinone oxidoreductase core subunit S2 and S7; *Ndufv3*, NADH:ubiquinone oxidoreductase subunit V3. (I and J) Enzymatic activity of complex I (I) and complex II (J) of the ETC in J2 macrophage cell lysate infected as in panel A. The data are expressed relative to uninfected macrophages, and error bars show the means ± the SD of four independent experiments. (K) Model showing the central metabolic pathways: glycolysis, TCA cycle, and OXPHOS. The arrows indicate how gene expression or metabolite levels changed following M. tuberculosis infection. *, *P* < 0.05; **, *P* < 0.01; ***, *P* < 0.005.

We analyzed the impact of M. tuberculosis Erdman infection on the expression of genes involved in central metabolic pathways using J2-immortalized murine macrophages and wild-type (WT) primary C57BL/6J mouse BMDM and resident peritoneal macrophages (RPM). The expression of hexokinase 2 (*Hk2*), the platelet isoform of phosphofructokinase (*Pfkp*), and glucose transporter 1 (*Glut1*), three rate-limiting enzymes in the glycolytic pathway (21, 22), was increased 24 h postinfection (p.i.) in J2 macrophages (Fig. 1A and B). The muscle and liver isoforms of phosphofructokinase (*Pfkm* and *Pfkl*, respectively) were decreased or unchanged (Fig. 1A). Mouse RPM also showed a 2.4-fold increase in *Hk2* at 24 h p.i. (see Fig. S1A in the supplemental material). Consistent with increased glycolytic gene expression, and in agreement with prior evidence that M. tuberculosis promotes glycolysis (3, 4, 23), secreted lactate levels were ∼50% higher in M. tuberculosis-infected J2 macrophages (Fig. 1C). Expression levels of the pentose phosphate pathway enzymes glucose-6 phosphate dehydrogenase (*G6pd*) and 6-phosphogluconate dehydrogenase (*Pgd*) were also increased (Fig. 1D). These results showed that M. tuberculosis induced the glycolytic and pentose phosphate pathways in macrophages.

10.1128/mBio.03140-20.1 FIG S1 M. tuberculosis induces metabolic transcriptional changes in mouse primary macrophages. (A) Gene expression in mouse resident peritoneal macrophages (*n* = 6 mice) infected with M. tuberculosis (MOI of 10; 24 h). The data are depicted as mean relative expression levels ± the SD. *Sirt3*, sirtuin 3; *Idh2*, isocitrate dehydrogenase 2; *Ndufv3*, NADH:ubiquinone oxidoreductase subunit V3; *Ndufs7*, NADH:ubiquinone oxidoreductase core subunit S7; *Hk2*, hexokinase 2. (B) Gene expression of *Idh2* in mouse BMDM infected with M. tuberculosis for 24 or 48 h. The data are expressed relative to uninfected conditions at 24 h and show the means ± the SD of three independent experiments. (C and D) Gene expression of the ETC complex I subunits, NADH:ubiquinone oxidoreductase core subunit S7 (*Ndufs7*) and NADH:ubiquinone oxidoreductase subunit V3 (*Ndufv3*), in mouse BMDM infected with M. tuberculosis at 24 h (C) and 48 h (D) p.i. The data are expressed relative to uninfected conditions and error bars show the means ± the SD of three independent experiments. (E) Gene expression of *Ndufs2* and *Ndufs7*, NADH:ubiquinone oxidoreductase core subunit S2 and S7, and *Ndufv3*, NADH:ubiquinone oxidoreductase subunit V3, in J2 macrophages infected with M. tuberculosis for 48 h. The data are expressed relative to uninfected conditions and analyzed as in panel A. (F and G) complex II, III, and IV protein subunits of the ETC in J2 macrophages infected with M. tuberculosis for 24 h (F) and 48 h (G) p.i. Gene expression was analyzed as described for panel A. *Sdha* and *Sdhd*, succinate dehydrogenase, subunits A and D; *Uqcrc2*, ubiqinol-cytochrome *c* reductase core protein II; *Cox5a*, cytochrome *c* oxidase subunit 5A. *, *P* < 0.05; **, *P* < 0.01; ***, *P* < 0.005. Download FIG S1, PDF file, 0.3 MB. Copyright © 2021 Smulan et al. 2021 Smulan et al. This content is distributed under the terms of the Creative Commons Attribution 4.0 International license.

To further assess metabolic alterations after infection, the transcriptional profiles of tricarboxylic acid (TCA) cycle and ETC components were analyzed. Expression of isocitrate dehydrogenase 2 (*Idh2*), a TCA cycle enzyme that catalyzes the decarboxylation of isocitrate to 2-oxoglutarate (15), was decreased by 40% in M. tuberculosis-infected J2 macrophages (Fig. 1E), RPM, and BMDM (see Fig. S1A and B). Activity of IDH2 is regulated through acetylation at lysine residues, where deacetylated IDH2 is more active (15). To analyze IDH2 acetylation in M. tuberculosis-infected J2 macrophages, acetylated proteins were immunoprecipitated, followed by immunoblotting with IDH2. While total IDH2 levels were decreased at 24 h p.i., the ratio of acetylated to total IDH2 was increased compared to uninfected controls (Fig. 1F), suggesting that M. tuberculosis alters IDH2 acetylation. Coinciding with changes in IDH2 protein, total isocitrate levels increased by 50% in infected J2 macrophages (Fig. 1G). These data indicated a block in the TCA cycle, mirroring the decrease in IDH observed in M1 macrophages activated by lipopolysaccharide (24).

The complex I subunit proteins NADH:ubiquinone oxidoreductase core subunits S2 and S7 (*Ndufs2* and *Ndufs7*), and NADH:ubiquinone oxidoreductase subunit V3 (*Ndufv3*) were transcriptionally downregulated in J2 macrophages at 24 h p.i. (Fig. 1H). Primary murine BMDM also showed decreased *Ndufs7* and *Ndufv3* (see Fig. S1C), while mouse RPM only showed a reduction in *Ndufv3* (see Fig. S1A). Reduced complex I subunit transcript expression was evident in both murine BMDM and J2 macrophages at 48 h p.i. (see Fig. S1D and E). Complex I enzymatic activity was reduced by ∼50% in J2 macrophage cell lysate following infection (Fig. 1I). Despite no change in expression of complex II, III, or IV subunits at 24 h p.i. (see Fig. S1F), complex II activity, or succinate dehydrogenase, was decreased by >50% in J2 macrophage cell lysate (Fig. 1J). Gene expression of the complex II subunits succinate dehydrogenase subunit A (*Sdha*) and subunit D (*Sdhd*), as well as the complex III subunit ubiquinol-cytochrome c reductase core protein 2 (*Uqcrc2*), were decreased by 48 h p.i. (see Fig. S1G). These results demonstrated blocks in the TCA cycle and ETC at SDH, which is the only enzyme participating in both processes. Collectively, as shown in Fig. 1K, our data revealed a shift toward aerobic glycolysis with downregulation of the TCA and ETC in M. tuberculosis-infected macrophages.

### *M. tuberculosis* provokes cellular ROS accumulation and mitochondrial stress.

**FIG 2:**
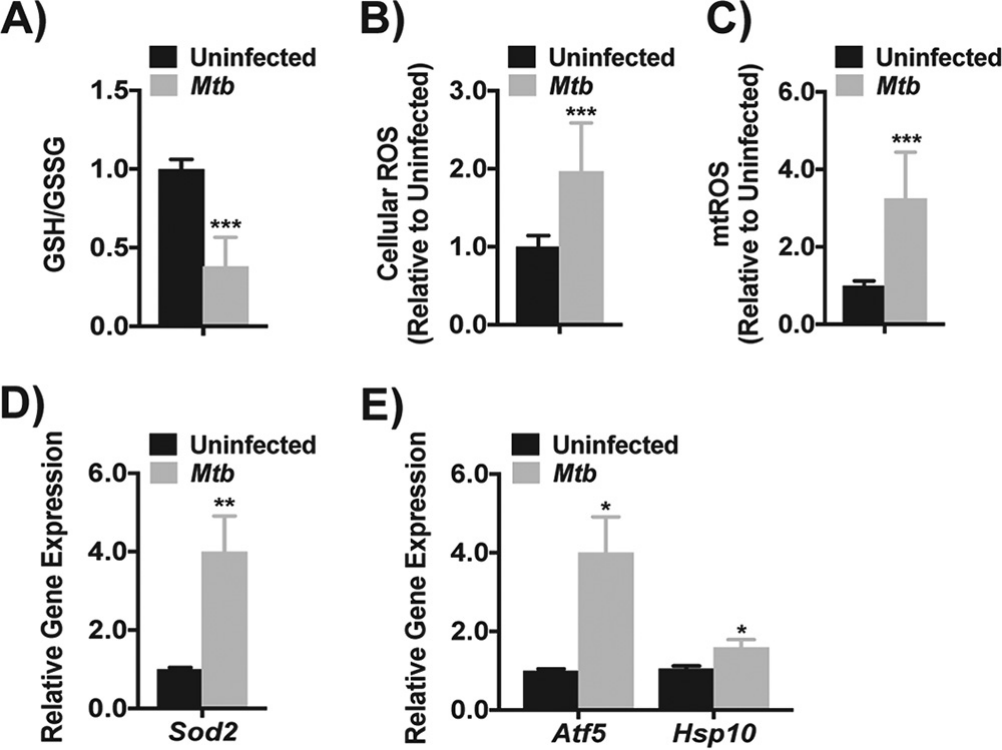
M. tuberculosis induces cellular ROS accumulation and mitochondrial stress. (A) Ratio of reduced (GSH) to oxidized (GSSG) glutathione in mouse J2 macrophage total cell lysate after M. tuberculosis infection (MOI of 10; 24 h). The data shown are relative to uninfected macrophages and are depicted as the means ± the SD of four independent experiments. (B and C) Analysis of cellular (B) and mitochondrial (mt) (C) reactive oxygen species (ROS) in J2 macrophages infected as in panel A. The data are expressed relative to uninfected macrophages and show the means ± the SD of three independent experiments. (D and E) Gene expression of superoxide dismutase 2 (*Sod2*) (D) and activating transcription factor 5 (*Atf5*) and heat shock protein 10 (*Hsp10*) (E) in J2 macrophages infected as in panel A. The data show the relative expression to uninfected macrophages, expressed as the means ± the SD of three independent experiments. *, *P* < 0.05; **, *P* < 0.01; ***, *P* < 0.005.

Electron leakage from ETC complex I is a main source of mtROS production (25). Reduced glutathione (GSH), an important ROS scavenger, is generated from glutathione disulfide (GSSG) in a reaction that requires NADPH generated by IDH2 (15, 16, 26). Based on the downregulation of IDH2 and ETC complex I subunit genes, we predicted that M. tuberculosis would induce oxidative stress and the mitochondrial unfolded protein response (UPR^mt^) in macrophages. We found that the GSH/GSSG ratio declined more than 2-fold in J2 macrophages at 24 h p.i. (Fig. 2A), due to lower GSH (see Fig. S2A) with GSSG levels unchanged (see Fig. S2B). Furthermore, total cellular ROS and mtROS nearly doubled after infection (Fig. 2B and C), matching prior studies (27, 28). Remarkably, ROS increased in M. tuberculosis-infected cells despite a 4-fold increase in *Sod2* gene that detoxifies superoxide (Fig. 2D) (29). Finally, gene expression of activating transcription factor 5 (*Atf5*) and heat shock protein 10 (*Hsp10*), mediators of the UPR^mt^ associated with mtROS accumulation (30–33), increased after infection (Fig. 2E). These results suggested that M. tuberculosis infection alters expression of TCA and ETC enzymes, leading to increased mtROS, combined with impaired ROS detoxification that together provoke mitochondrial stress.

10.1128/mBio.03140-20.2 FIG S2 M. tuberculosis alters cellular redox in macrophages. (A and B) Analysis of reduced (GSH) (A) and oxidized (GSSG) (B) glutathione in mouse J2 macrophage total cell lysate infected with M. tuberculosis (MOI of 10) for 24 h. Error bars show the means ± the SD of four independent experiments. *, *P* < 0.05. Download FIG S2, PDF file, 0.04 MB. Copyright © 2021 Smulan et al. 2021 Smulan et al. This content is distributed under the terms of the Creative Commons Attribution 4.0 International license.

### SIRT3 downregulation in *M. tuberculosis*-infected macrophages is mediated by TLR2.

**FIG 3:**
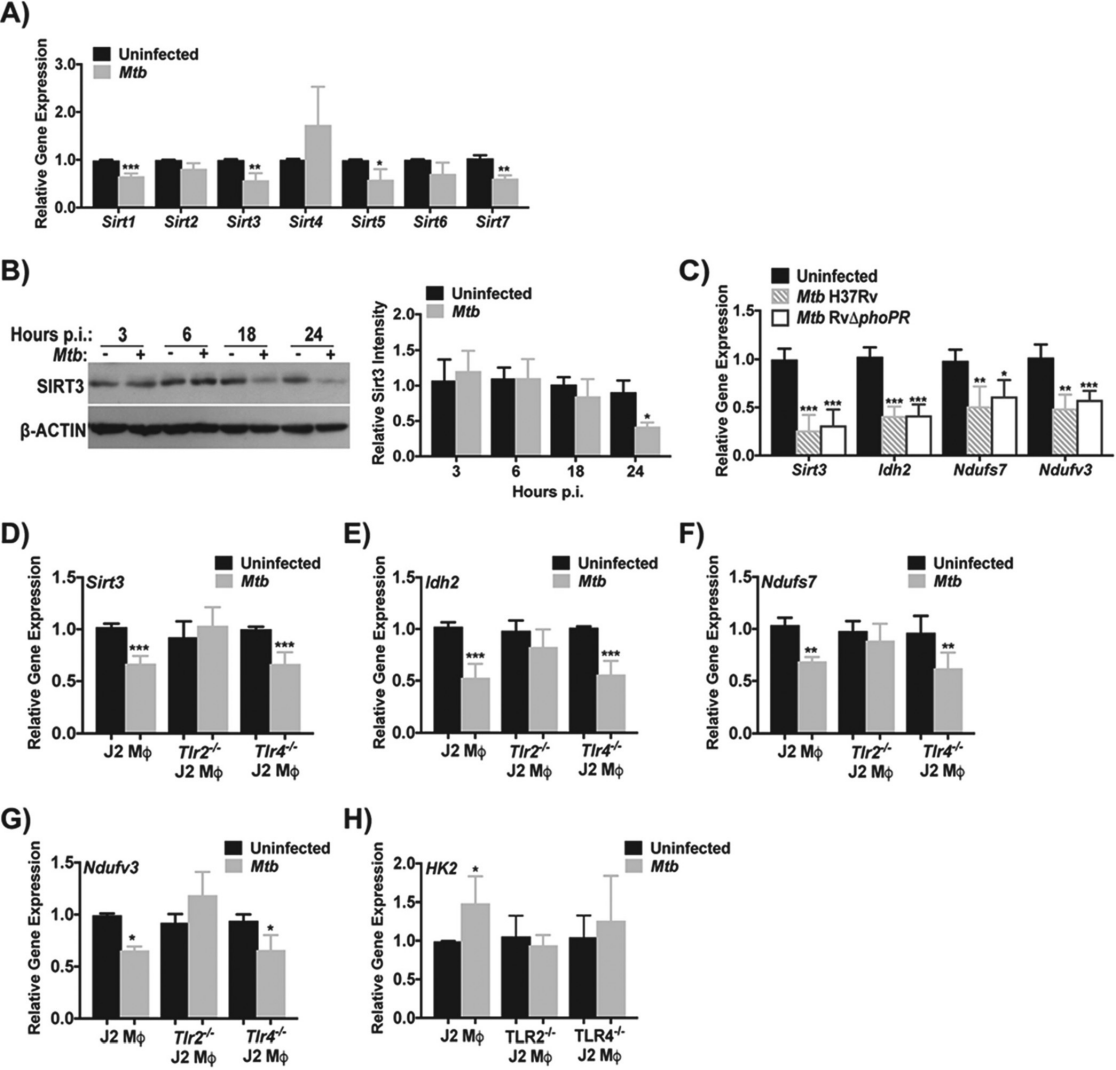
M. tuberculosis-mediated downregulation of SIRT3 is TLR2-Dependent. (A) Gene expression of the mammalian sirtuins (*Sirt1-7*) in J2 macrophage infected with M. tuberculosis Erdman (MOI of 10) for 24 h. Genes are expressed relative to uninfected macrophages. Plots show the means ± the SD from four independent experiments. (B, left) Representative immunoblot showing SIRT3 and β-actin in J2 macrophage total cell lysate after M. tuberculosis Erdman infection for 3, 6, 18, and 24 h. (Right) Densitometry analysis of immunoblots. The data are normalized to β-actin and expressed relative to uninfected conditions. Plots show the means ± the SD of three independent experiments. (C) Gene expression of *Sirt3*, *Idh2*, *Ndufs7*, and *Ndufv3* in mouse BMDM infected with H37RV or RVΔ*phoPR* (MOI of 10) for 24 h. Genes are expressed relative to uninfected macrophages and plots show the means ± the SD from three independent experiments. (D to H) Gene expression of sirtuin 3 (*Sirt3*) (D), isocitrate dehydrogenase 2 (*Idh2*) (E), NADH:ubiquinone oxidoreductase core subunit S7 (*Ndufs7*) (F), NADH:ubiquinone oxidoreductase subunit V3 (*Ndufv3*) (G), and hexokinase-2 (*Hk2*) (H) in wild type, *Tlr2^−/−^*, and *Tlr4^−/−^* J2 macrophages with identical conditions and analysis as in panel A. *, *P* < 0.05; **, *P* < 0.01; ***, *P* < 0.005.

To assess the relationship between metabolism and mtROS induction after M. tuberculosis infection, we investigated the expression of SIRT3, an enzyme that is known to regulate mitochondrial metabolism and cellular redox homeostasis (14–16, 19, 34). Sirtuin activity depends on NAD^+^ and we previously reported that macrophage NAD^+^ pools decrease following infection (27, 35). Speculating that M. tuberculosis modulates sirtuin activity by a combination of cofactor depletion and transcriptional suppression, we measured mRNA levels of the mammalian sirtuins in J2 macrophages. The expression of *Sirt1*, *Sirt3*, *Sirt5*, and *Sirt7* were all decreased at 24 h p.i. (Fig. 3A) and this was validated in BMDM (see Fig. S3A and B). The decrease in *Sirt1* agreed with our previous study highlighting a role of SIRT1 in TB pathogenesis (35). The decrease in mitochondrial sirtuins (SIRT3 and SIRT5) indicated a role for these deacetylases in the host mitochondrial response to M. tuberculosis. Reduced *Sirt3* mRNA levels were also observed in mouse RPM at 24 h p.i. (see Fig. S1A). To explore the kinetics of *Sirt3* modulation, we measured mRNA and protein levels at 3, 6, 18, and 24 h p.i. *Sirt3* mRNA was significantly decreased starting at 3 h and remained down through 24 h p.i. (see Fig. S3C), while protein levels only decreased at 24 h p.i. (Fig. 3B). SIRT3 protein levels also decreased in M. tuberculosis-infected BMDM (see Fig. S3D). To address the role of M. tuberculosis virulence functions in SIRT3 downregulation, we compared H37Rv with an isogenic strain lacking the PhoPR regulator that controls both type VII secretion via ESX1 and virulence-associated lipid production (36). This attenuated mutant downregulated *Sirt3*, *Idh2*, *Ndufs7*, and *Ndufv3* in BMDM to levels identical to that of H37Rv-infected BMDM (Fig. 3C), suggesting that transcriptional regulation is independent of these bacterial virulence factors.

10.1128/mBio.03140-20.3 FIG S3 M. tuberculosis-mediated downregulation of SIRT3 is TLR2-dependent. (A) Gene expression of mammalian sirtuins (*Sirt1*, *Sirt2*, and *Sirt4-7*) was analyzed in wild-type (WT) and *Sirt3^−/−^* BMDM infected with M. tuberculosis (MOI of 10; 24 h). Genes are expressed relative to uninfected WT BMDM and show the means ± the SD of three independent experiments. (B) Sirtuin 3 gene (*Sirt3*) expression in mouse BMDM infected with M. tuberculosis 24 or 48 h. The data are shown relative to uninfected macrophages at 24 h p.i., and error bars show the means ± the SD from three independent experiments. (C) Analysis of *Sirt3* gene expression in J2 macrophages infected with M. tuberculosis for 3, 6, 18, and 24 h. Genes are expressed as relative to uninfected macrophages and show the means ± the SD of three independent experiments. (D, left) Representative immunoblot showing protein expression of SIRT3 and β-ACTIN in primary mouse BMDM total cell lysate infected with M. tuberculosis as in panel A. (Right) Densitometry analysis of immunoblots. The data are normalized to β-actin and expressed relative to uninfected conditions. Plots show the means ± the SD of three independent experiments. (E to H) Gene expression of sirtuin 3 (*Sirt3*) (E), the ETC complex I subunits, NADH:ubiquinone oxidoreductase core subunit S7 (*Ndufs7*) and NADH:ubiquinone oxidoreductase subunit V3 (*Ndufv3*) (F), isocitrate dehydrogenase 2 (*Idh2*) (G), and hexokinase-2 (*Hk2*) (H) in wild-type (WT) and *Myd88^−/−^/Trif^−/−^* BMDM infected as described for panel A. Genes are expressed relative to uninfected WT BMDM and expressed as the means ± the SD of four independent experiments. *, *P* < 0.05; **, *P* < 0.01; ***, *P* < 0.005. Download FIG S3, PDF file, 0.5 MB. Copyright © 2021 Smulan et al. 2021 Smulan et al. This content is distributed under the terms of the Creative Commons Attribution 4.0 International license.

SIRT3 is reduced in lung homogenate and alveolar macrophages treated with the Toll-like receptor 4 (TLR4) ligand lipopolysaccharide (37), suggesting that Toll signaling might mediate SIRT3 downregulation by M. tuberculosis. To test that hypothesis, we challenged *Myd88^−/−^/Trif^−/−^* double knockout BMDM lacking signal adapters essential for all TLR signaling (38). As predicted, infection of WT but not *Myd88^−/−^/Trif^−/−^* BMDM showed more than 50% reduction in *Sirt3* by 24 h p.i. (see Fig. S3E). Next, we measured *Sirt3* levels in *Tlr2^−/−^* or *Tlr4^−/−^* J2-immortalized macrophages, finding decreased expression in M. tuberculosis-infected WT and *Tlr4^−/−^* J2 macrophages but not in *Tlr2^−/−^* J2 macrophages (Fig. 3D). Like *Sirt3*, the transcriptional regulation of *Idh2*, *Ndufs7*, *Ndufv3*, and *Hk2* also depended on TLR2; however, TLR4 was also important for *Hk2* regulation (Fig. 3E to H; see also Fig. S3F to H). Altogether, these results showed that recognition of M. tuberculosis by TLR2 was required for the transcriptional regulation of *Sirt3* and its downstream targets.

### SIRT3 regulates genes involved in central metabolism and *M. tuberculosis*-induced mtROS accumulation and cell death.

**FIG 4:**
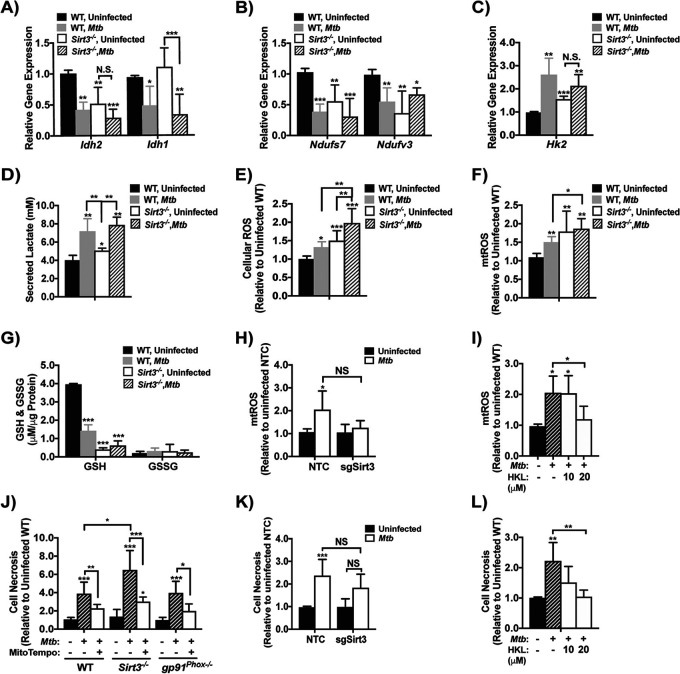
SIRT3 regulates genes involved in central metabolism and is important for M. tuberculosis-induced ROS accumulation. (A to C) Gene expression of isocitrate dehydrogenase 1 and 2 (*Idh1* and *Idh2*) (A), NADH:ubiquinone oxidoreductase core subunit S7 (*Ndufs7*) and NADH:ubiquinone oxidoreductase subunit V3 (*Ndufv3*) (B), and hexokinase 2 (*Hk2*) (C) in wild type (WT) and *Sirt3^−/−^* BMDM infected with M. tuberculosis (MOI of 10) for 24 h. The data are expressed relative to uninfected WT BMDM and show the means ± the SD of four independent experiments. (D) Secreted lactate levels from WT and *Sirt3^−/−^* BMDM infected with M. tuberculosis as described previously (A). Error bars show the means ± the SD of four independent experiments. (E and F) Analysis of cellular (E) and mitochondrial (mt) (F) reactive oxygen species (ROS) in WT and *Sirt3^−/−^* BMDM with infection conditions and analysis as in panel A of three independent experiments. (G) Analysis of reduced (GSH) and oxidized (GSSG) glutathione in WT or *Sirt3^−/−^* BMDM total cell lysate infected with M. tuberculosis as in panel A. Plots show the means ± the SD of four independent experiments. (H) Analysis of mtROS in J2 macrophages expressing single guide RNA for Sirt3 (sgSirt3) or the nontemplate control (NTC) with identical infection conditions as described for panel A. The data are expressed relative to uninfected macrophages expressing the NTC and show the means ± the SD of four independent experiments. (I) Analysis of mtROS in WT BMDM treated with 10 or 20 μM of the SIRT3 activator, honokiol (HKL), and infected as in panel A. mtROS is expressed relative to uninfected, untreated WT BMDM, and the means ± the SD of four independent experiments are shown. (J) Cell necrosis of WT, *Sirt3^−/−^*, and *gp91^phox–/–^* BMDM treated with the ROS scavenger MitoTEMPO (500 μM) or vehicle and infected as in panel A. The data are expressed relative to uninfected, untreated WT BMDM and show the means ± the SD of four independent experiments. (K) Cell necrosis of J2 macrophages expressing sgSirt3 or the NTC with identical infection conditions as in panel A. The data are expressed relative to uninfected NTC-expressing macrophages and show the means ± the SD of four independent experiments. (L) Cell necrosis of WT BMDM treated with HKL (10 or 20 μM) and infected as in panel A. The data are expressed relative to uninfected, untreated WT BMDM and show the means ± the SD of four independent experiments. *, *P* < 0.05; **, *P* < 0.01; ***, *P* < 0.005.

Since SIRT3 regulates the expression and activity of key enzymes involved in central metabolism including IDH2 (19, 39), we hypothesized that metabolic reprogramming by M. tuberculosis (Fig. 1) results from SIRT3 modulation. The basal level of *Idh2* mRNA in uninfected *Sirt3^−/−^* BMDM was 48% lower than in the WT, while the cytosolic isoform *Idh1* was unchanged (Fig. 4A). Infection with M. tuberculosis produced a significant reduction in *Idh2* in WT BMDM with no reduction in *Sirt3^−/−^* BMDM (Fig. 4A). However, *Idh1* was reduced in both WT and *Sirt3^−/−^* BMDM by 24 h p.i. (Fig. 4A). Similar trends were observed for *Ndufs7* and *Ndufv3* (Fig. 4B). Silencing *Sirt3* in uninfected J2 macrophages (50% reduction; see Fig. S4A) was accompanied by decreased expression of *Idh2*, *Ndusf7*, and *Ndufv3* (see Fig. S4B to D) that was not further reduced by M. tuberculosis, consistent with results using *Sirt3^−/−^* BMDM. Together, these findings indicated that downregulation of *Idh2* and the ETC complex I subunits *Ndufs7* and *Ndufv3* following M. tuberculosis infection reflected SIRT3-dependent regulation.

10.1128/mBio.03140-20.4 FIG S4 SIRT3 regulates genes involved in central metabolism. (A to D) J2 macrophages were transfected with silencing RNA for *Sirt3* (si*Sirt3*) or the scrambled control sequence 24 h before infection with M. tuberculosis (MOI of 10; 24 h). The gene expression of *Sirt3* (A), *Idh2* (B), *Ndufs7* (C), and *Ndufv3* (D) was analyzed, and genes are shown relative to uninfected macrophages expressing the scrambled control. The data are depicted as the means ± the SD of three independent experiments. *Sirt3*, sirtuin 3; *Idh2*, isocitrate dehydrogenase 2; *Ndufs7*, NADH:ubiquinone oxidoreductase core subunit S7; *Ndufv3*, NADH:ubiquinone oxidoreductase subunit V3. (E) Gene expression of phosphofructokinase, platelet (*Pfkp*), muscle (*Pfkm*), and liver (*Pfkl*) types was analyzed in WT and *Sirt3^−/−^* BMDM with infection conditions as described for panel A. Genes are shown relative to uninfected WT BMDM and expressed as the means ± the SD of three independent experiments. (F) Analysis of reduced (GSH) to oxidized (GSSG) glutathione in WT and *Sirt3^−/−^* BMDM total cell lysate with infection conditions as described for panel A. The data are expressed relative to uninfected WT BMDM and show the means ± the SD of four independent experiments. (G) *Sirt3* gene expression in J2 macrophages expressing single guide RNA for Sirt3 (sgSirt3) or the nontemplate control (NTC). The data are expressed relative to NTC and show the means ± the SD of three independent experiments. (H) Gene expression of activating transcription factor 5 (*Atf5*) was analyzed in WT and *Sirt3^−/−^* BMDM with infection conditions as described for panel A. The data are expressed relative to uninfected WT BMDM and shown as the means ± the SD of three independent experiments. (I) Cell viability of WT, *Sirt3^−/−^* and *gp91^Phox–/–^* BMDM treated with the ROS scavenger MitoTEMPO (500 μM) or vehicle and infected as described for panel A. The data are expressed relative to uninfected, untreated WT BMDM and show the means ± the SD of five independent experiments. (J) Mitochondrial ROS (mtROS) analysis in WT, *Sirt3^−/−^*, and *gp91^phox–/–^* BMDM treated with the ROS scavenger MitoTEMPO (100, 250, or 500 μM) or vehicle and infected as in panel A. The data are expressed relative to uninfected, untreated WT BMDM and show the means ± the SD of three independent experiments. *, *P* < 0.05; **, *P* < 0.01; ***, *P* < 0.005. Download FIG S4, PDF file, 0.5 MB. Copyright © 2021 Smulan et al. 2021 Smulan et al. This content is distributed under the terms of the Creative Commons Attribution 4.0 International license.

To investigate the role of SIRT3 in regulating glycolytic enzymes, gene expression of HK2 and PFK-P, -M, and -L was analyzed in WT and *Sirt3^−/−^* BMDM. Expression of *Hk2* was 1.6-fold higher in uninfected *Sirt3^−/−^* BMDM compared to WT (Fig. 4C) and was not further increased by M. tuberculosis, suggesting that the infection-induced increase of *Hk2* in WT macrophages resulted from SIRT3 downregulation (Fig. 4C). Unlike *Hk2*, basal expression of the glycolytic enzymes *Pfkp*, *Pfkm*, and *Pfkl* was similar between uninfected *Sirt3^−/−^* and WT cells, but the expression of *Pfkl* was increased in WT and *Sirt3^−/−^* BMDM by 24 h p.i. (see Fig. S4E). Secreted lactate from uninfected *Sirt3^−/−^* BMDM was 20% higher than in WT controls (Fig. 4D), indicating a bias of *Sirt3^−/−^* BMDM toward glycolysis under basal conditions. M. tuberculosis significantly increased secreted lactate in both WT and *Sirt3^−/−^* BMDM compared to uninfected WT controls (Fig. 4D). Together, these data supported an infection-induced metabolic shift toward glycolysis in primary and J2 macrophages due, at least in part, to reduced SIRT3 expression.

Since depletion of core ETC complex I subunits or treatment with rotenone increase ROS production (40, 41), we considered that downregulation of complex I subunits and reduced complex I enzymatic activity in M. tuberculosis-infected macrophages (Fig. 1H and I) contributed to the increased ROS production that others reported (27, 42), and we confirmed (Fig. 2B and C). To demonstrate a mechanistic link between SIRT3 and ROS, we measured cellular ROS levels in WT and *Sirt3^−/−^* BMDM. Infected WT BMDM showed increased total cellular ROS (Fig. 4E). Basal ROS levels in *Sirt3^−/−^* BMDM were comparable to levels in infected WT cells (Fig. 4E). Infection of *Sirt3^−/−^* BMDM resulted in a further 1.5-fold increase of ROS compared to infected WT cells (Fig. 4E). Uninfected *Sirt3^−/−^* BMDM demonstrated higher mtROS levels compared to WT cells (Fig. 4F), which was not further increased by M. tuberculosis infection (Fig. 4F). Since IDH2 is a major contributor of NADPH required for GSH production (15, 16) and since we identified SIRT3-dependent modulation of IDH2 (Fig. 4A), we reasoned that GSH production also depends on SIRT3. Uninfected *Sirt3^−/−^* mice showed a >75% decrease in GSH compared to uninfected WT BMDM (Fig. 4G), while GSSG remained unchanged (Fig. 4G), reducing the GSH/GSSG ratio by ∼80% (see Fig. S4F). Infection of *Sirt3^−/−^* BMDM did not cause a further decline of GSH or the GSH/GSSG ratio compared to uninfected *Sirt3^−/−^* or infected WT BMDM (Fig. 4G; see also Fig. S4F). Lastly, we measured mtROS in macrophages with genetic overexpression of SIRT3 and in WT BMDM treated with the SIRT3 activator Honokiol (43). Overexpression of SIRT3 (8-fold induction; see Fig. S4G) and Honokiol treatment both prevented M. tuberculosis-induced mtROS accumulation (Fig. 4H and I). These results demonstrated the importance of SIRT3 in M. tuberculosis-induced mtROS accumulation.

We next assessed the role of SIRT3 in M. tuberculosis-induced UPR^mt^ (Fig. 2E) by measuring *Atf5* in *Sirt3^−/−^* BMDM. As shown in Fig. S4H, basal *Atf5* levels were similar in uninfected WT and *Sirt3^−/−^* BMDM despite higher total cellular ROS and mtROS in the latter (Fig. 4E and F). In unstressed *Sirt3^−/−^* cells, other SIRTs presumably compensated to prevent UPR^mt^. Infection of WT BMDM with M. tuberculosis raised *Atf5* mRNA levels 1.6-fold, while this was increased 2.0-fold in *Sirt3^−/−^* BMDM (see Fig. S4H). These data supported a role for SIRT3 downregulation in the accumulation of ROS and consequently mitochondrial stress after M. tuberculosis infection.

We next questioned whether mtROS accumulation following SIRT3 modulation contributed to M. tuberculosis-induced macrophage cell death. Cell necrosis was increased ∼4-fold in M. tuberculosis-infected WT BMDM, with a further increase in *Sirt3^−/−^* BMDM (Fig. 4J). Analysis of cell viability showed similar trends (see Fig. S4I), indicating that *Sirt3^−/−^* cells were more susceptible to M. tuberculosis-induced cell death. Treatment with the mitochondria-specific superoxide scavenger MitoTEMPO (500 μM) prevented M. tuberculosis-induced mtROS accumulation in WT BMDM and partially prevented accumulation in *Sirt3^−/−^* BMDM (see Fig. S4J), while rescuing WT macrophages from necrosis and restoring cellular viability (Fig. 4J; see also Fig. S4I). Viability and necrosis were only partially rescued in *Sirt3^−/−^* BMDM at 24 h p.i. (Fig. 4J; see also Fig. S4I). Furthermore, SIRT3 overexpression or activation with Honokiol partially rescued cells from cell necrosis (Fig. 4K and L), indicating a role for SIRT3 in mtROS-induced cell death.

Multiple sources contribute to cellular ROS, including ETC complex I, and NADPH oxidase (44, 45). To determine whether NADPH oxidase contributed to cytotoxic ROS accumulation, NADPH oxidase-deficient *gp91^phox–/–^* BMDM were challenged with M. tuberculosis and viability was assessed. Mirroring WT BMDM, M. tuberculosis induced cell death and reduced cell viability in *gp91^phox–/–^* BMDM (Fig. 4J; see also Fig. S4I). Treatment with MitoTEMPO to limit mtROS accumulation in *gp91^phox–/–^* BMDM (see Fig. S4J), rescued *gp91^phox–/–^* BMDM from cell necrosis and restored cell viability similar to WT BMDM (Fig. 4J; see also Fig. S4I). These results indicated that mtROS but not ROS derived from NADPH oxidase played a substantial role in M. tuberculosis-induced macrophage cell death and that the increased susceptibility of *Sirt3^−/−^* BMDM to cell death was due in part to increased mtROS.

### SIRT3 is required for protection against *M. tuberculosis in vivo*.

**FIG 5:**
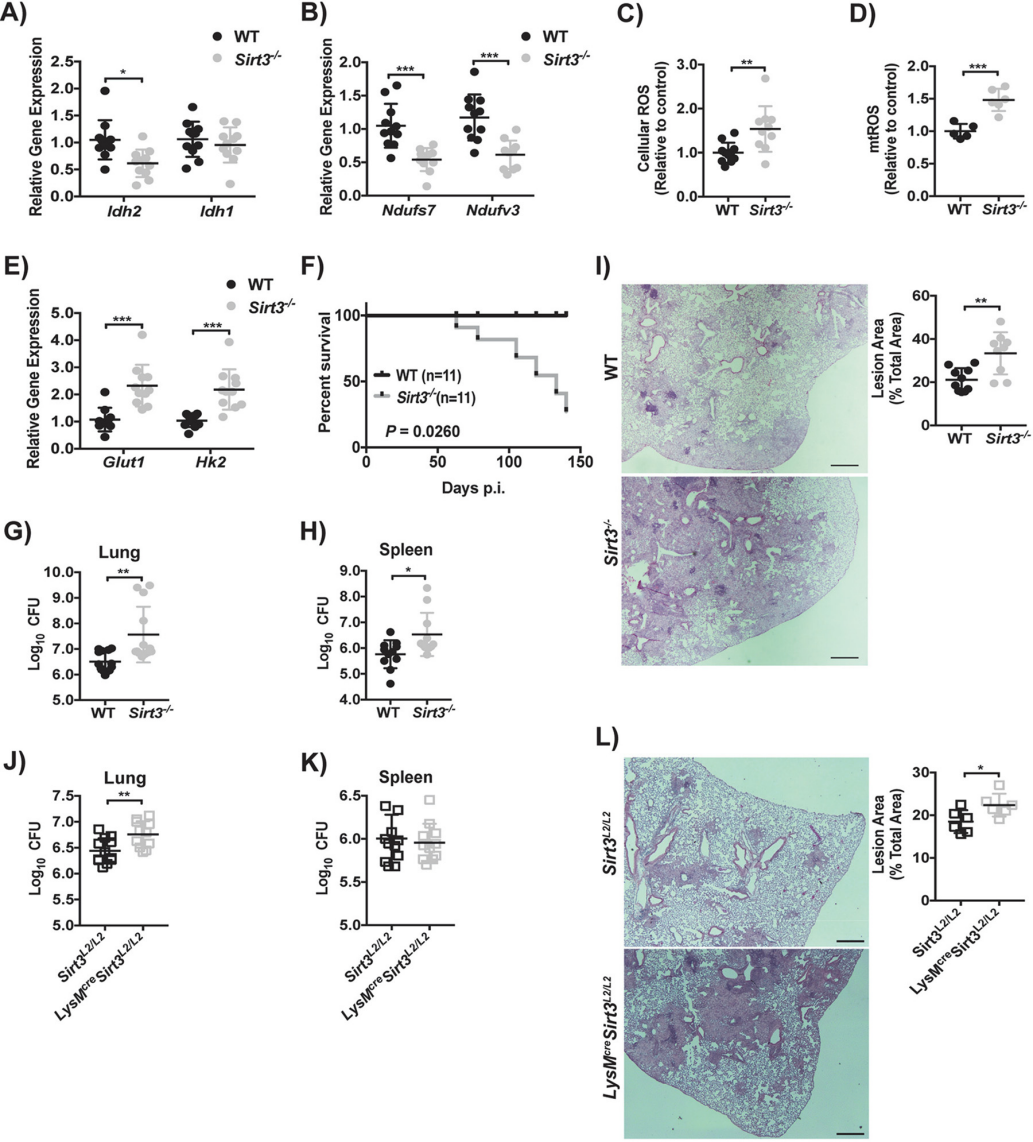
SIRT3 regulates metabolic gene expression *in vivo* and protects host against M. tuberculosis infection. (A and B) Wild-type (WT) and *Sirt3^−/−^* mice (*n* = 11 per group) were infected with M. tuberculosis by aerosol and CD11b^+^ lung leukocytes were isolated at 16 weeks p.i. The gene expression of *Idh2* and *Idh1* (A) and *Ndufs7* and *Ndufv3* (B) was analyzed in isolated cells. The data are expressed relative to WT and are shown as the means ± the SD of two independent experiments. *Idh*, isocitrate dehydrogenase; *Ndufs7*, NADH:ubiquinone oxidoreductase core subunit S7; *Ndufv3*, NADH:ubiquinone oxidoreductase subunit V3. (C) Analysis of cellular reactive oxygen species (ROS) in isolated CD11b^+^ lung leukocytes under identical conditions as in panel A. The data are expressed relative to the WT and shown as the means ± the SD of two independent experiments. (D) Analysis of mitochondrial ROS (mtROS) in isolated CD11b^+^ lung leukocytes from WT and *Sirt3^−/−^* mice (*n* = 6 per group) infected as in panel A. The data are expressed relative to WT and shown as the means ± the SD. (E) Gene expression of glucose transporter 1 (*Glut1*) and hexokinase 2 (*Hk2*) in isolated CD11b^+^ lung leukocytes under identical conditions and analysis as described for panel A. (F) Survival of M. tuberculosis-infected WT and *Sirt3^−/−^* mice (*n* = 11) infected as in panel A. Mice were monitored for 20 weeks p.i., when all surviving mice were sacrificed. (G and H) CFU in lung (G) and spleen (H) homogenates from WT and *Sirt3^−/−^* mice (*n* = 11 to 13 per group) infected as in panel A. Data include CFU from 9 to 20 weeks p.i., expressed as means ± the SD of two independent experiments. (I) Representative lung histopathology by hematoxylin and eosin staining and analysis of lesion area from WT and *Sirt3^−/−^* mice (*n* = 9 to 10 per group) infected with M. tuberculosis as in panel A. The size of the lesion area was analyzed using ImageJ software. Scale bar, 500 μm. The data show the means ± the SD of two independent experiments. (J and K) CFU in lung (J) and spleen (K) homogenates from *Sirt3^L2/L2^* and *LysM^cre^Sirt^L2/L2^* mice (*n* = 11 to 12 per group) infected as in panel A. The data include CFU from 8 weeks p.i., expressed as the means ± the SD of two independent experiments. (L) Representative lung histopathology stained and analyzed as in panel I from *Sirt3^L2/L2^* and *LysM^cre^Sirt^L2/L2^* mice (*n* = 6 per group) infected as in panel A. Scale bar, 500 μm. The data show the means ± the SD. *, *P* < 0.05; **, *P* < 0.01; ***, *P* < 0.005.

Since our *in vitro* data identified SIRT3 as a regulator of metabolic reprogramming in M. tuberculosis-infected macrophages *in vitro*, we sought to determine whether this was recapitulated *in vivo* and whether it was associated with altered TB susceptibility. WT and *Sirt3^−/−^* mice were challenged with 100 CFU of M. tuberculosis Erdman and the expression of the TCA cycle, glycolytic and ETC subunit genes in CD11b^+^ lung leukocytes was measured at 16 weeks p.i. The levels of *Idh2* mRNA were ∼40% lower in CD11b^+^ lung leukocytes from infected *Sirt3^−/−^* mice compared to WT mice, while *Idh1* showed no change (Fig. 5A). This demonstrated regulation of *Idh2* expression by SIRT3, which has not been described before. Further matching the *in vitro* data (Fig. 4B), the expression of *Ndufs7* and *Ndufv3* was downregulated in CD11b^+^ lung leukocytes from *Sirt3^−/−^* mice (Fig. 5B). Correlating with the changes in complex I subunits and *Idh2*, CD11b^+^ lung leukocytes from infected *Sirt3^−/−^* mice had 1.7- and 1.5-fold increases in cellular and mtROS, respectively, compared to WT mice (Fig. 5C and D). The expression of *Glut1* and *Hk2* was increased 2-fold in CD11b^+^ lung leukocytes from infected *Sirt3^−/−^* mice (Fig. 5E), which might also contribute to ROS accumulation (27). These results were consistent with a role for SIRT3 as a regulator of genes involved in glycolysis, TCA, and ETC *in vivo*, and they confirmed perturbations of SIRT3 mediated ROS accumulation during M. tuberculosis infection *in vitro*.

Based on our *in vitro* data and reports on the Warburg effect in TB, we predicted that reduced SIRT3 expression would contribute to excessive inflammation and a permissive environment for M. tuberculosis in the lung. WT and *Sirt3^−/−^* mice were challenged with M. tuberculosis by aerosol and monitored up to 20 weeks p.i. *Sirt3^−/−^* mice showed increased mortality starting at 9 weeks p.i. and reaching 50% by 17 weeks (Fig. 5F). Lung and spleen CFU were not different in WT or *Sirt3^−/−^* mice at 2 and 4 weeks p.i. (see Fig. S5A and B), but the bacterial burden was significantly higher in lungs and spleens of *Sirt3^−/−^* mice starting at 9 weeks p.i. compared to WT mice (Fig. 5G and H). The lung tissue area involved with TB immune pathology was also greater in *Sirt3^−/−^* than in WT mice (Fig. 5I). Similar to *Sirt3^−/−^* mice, the bacterial load was increased in the lungs, but not spleens, of M. tuberculosis-infected *LysM^cre^Sirt3^L2/L2^* mice compared to littermate controls at 8 weeks p.i. (Fig. 5J and K). By 16 weeks p.i., both the lungs and the spleens of *LysM^cre^Sirt3^L2/L2^* mice showed a greater bacterial burden compared to littermate controls (see Fig. S5C and D). Infected *LysM^cre^Sirt3^L2/L2^* mice also showed greater lesion area compared to littermate controls (Fig. 5L). Taken together, our data illustrated a novel role for SIRT3 in regulating macrophage mitochondrial and glycolytic gene expression during M. tuberculosis infection *in vitro* and in TB disease *in vivo*.

10.1128/mBio.03140-20.5 FIG S5 Bacterial burden in whole body *Sirt3^−/−^* mice and macrophage-specific *Sirt3^−/−^* mice infected with M. tuberculosis. (A and B) Wild-type (WT) and *Sirt3^−/−^* mice (*n* = 5 per group) were infected with M. tuberculosis Erdman by the aerosol route, and the CFU in lung (A) and spleen (B) homogenates were analyzed at 14 and 28 days p.i. The data are expressed as the means ± the SD. (C and D) CFU in lung (C) and spleen (D) homogenates from M. tuberculosis-infected female *Sirt3^L2/L2^* and *LysM^cre^Sirt^L2/L2^* mice (*n* = 4 to 6 per group) were analyzed at 16 weeks p.i. The data are expressed as the means ± the SD. *, *P* < 0.05. Download FIG S5, PDF file, 0.3 MB. Copyright © 2021 Smulan et al. 2021 Smulan et al. This content is distributed under the terms of the Creative Commons Attribution 4.0 International license.

## DISCUSSION

Several studies reported that M. tuberculosis shifts mitochondrial bioenergetic metabolism toward aerobic glycolysis in human and murine macrophages (2–5). We confirmed and extended those findings by demonstrating a role for SIRT3 as an upstream regulator of that response. We show that SIRT3 mRNA and protein levels decreased in M. tuberculosis*-*infected macrophages, along with reduced expression of mitochondrial metabolic pathway enzymes, including the SIRT3 targets IDH2, and ETC complex I subunits. This resulted in isocitrate accumulation, reduced complex I and II activity, a lower GSH/GSSG ratio, and increased mtROS. The downregulation of *Idh2*, *Ndusf7*, and *Ndufv3* was SIRT3 dependent, underscoring the importance of SIRT3 in regulating basal mitochondrial metabolism and its dysregulation following M. tuberculosis infection. Our data support a model where M. tuberculosis triggers TLR2, resulting in downregulation of SIRT3 and its target genes leading to perturbation of mitochondrial bioenergetics and consequently mtROS accumulation and cell death.

Kim et al. reported that a very high intranasal dose of M. tuberculosis H37Rv (3 × 10^4^ CFU) provokes rapid mortality (within several days) and increased bacterial burden in *Sirt3^−/−^* mice (20). In our study, a low-dose aerosol infection with M. tuberculosis Erdman also increased the mortality of *Sirt3^−/−^* mice compared to WT controls. The host-protective role of SIRT3 after infection appears to be restricted to M. tuberculosis, rather than a generalized phenomenon, since *Sirt3*^−/−^ mice do not have an altered bacterial burden and survival when subjected to endotoxemia, Escherichia coli peritonitis, Klebsiella pneumoniae pneumonia, listeriosis, or candidiasis (46). We show for the first time the importance of macrophage-specific SIRT3 expression as the bacterial load within lungs from *LysM^cre^Sirt3^L2/L2^* mice was increased, similar to *Sirt3^−/−^* mice. Interestingly, bacterial burden within lungs of *Sirt3^−/−^* mice was higher starting at 9 weeks p.i., while bacterial load was similar between *Sirt3^−/−^* and WT mice at 2 or 4 weeks p.i. A recent study by Huang et al. showed that M. tuberculosis-infected alveolar and interstitial macrophages have distinct metabolic states which effects bacterial growth. Interstitial macrophages were more dependent on glycolysis than OXPHOS and showed restrictive bacterial growth compared to alveolar macrophages (47). Macrophages from *Sirt3^−/−^* mice may undergo a shift toward glycolysis, which could limit bacterial growth at the earlier time points, and as infection persists, mtROS accumulates leading to mitochondrial stress resulting in an environment detrimental for the macrophage, leading to the increased bacterial load and spread. Future work will focus on understanding the mechanisms of increased susceptibility of *Sirt3^−/−^* mice to M. tuberculosis.

We further extended on the study by Kim et al. (20) by showing TLR2-dependent transcriptional downregulation of *Sirt3* and its target genes—*Ndusf7*, *Ndufv3*, and *Idh2*—in M. tuberculosis-infected macrophages. We show that SIRT3 downregulation increased mtROS production and lowered cellular GSH, provoking oxidative stress, UPR^mt^, and macrophage cell death. Our data suggest that elevated mtROS after infection results from downregulation of SIRT3 and consequently NDUFS7 and NDUFV3. However, decreased SDH activity (Fig. 1J), which may increase succinate levels (48, 49), and NAD^+^ hydrolysis by the tuberculosis necrotizing toxin (28, 50) could also contribute. We found no role for ROS generated by NADPH oxidase in M. tuberculosis-induced macrophage cytolysis. Overall, these results add new understanding of complex I regulation by SIRT3, demonstrating SIRT3-dependent regulation of *Ndufs7* and *Ndufv3* transcripts *in vitro* and *in vivo*.

Mitochondrial matrix-associated IDH2 is a target of SIRT3, which increases enzymatic activity by deacetylation (15). We found that SIRT3 modulation by M. tuberculosis leads to decreased IDH2 transcript and protein levels and increased levels of isocitrate. The link between IDH2 and SIRT3 was supported by finding lower basal *Idh2* mRNA levels in uninfected *Sirt3^−/−^* BMDM and in CD11b^+^ lung leukocytes from infected *Sirt3^−/−^* mice compared to WT controls. As IDH2 consumes NADP^+^ and generates NADPH that maintains GSH, downregulation of IDH2 also compromises ROS detoxification (26, 51, 52). SIRT3 was previously shown to regulate cellular redox by altering IDH2 activity (16). While we did not measure NADPH levels in M. tuberculosis-infected macrophages, a prior study reported decreased NADPH levels in lungs of mice infected with H37Rv (53), and we found SIRT3-dependent lowering of GSH in infected macrophages (Fig. 4G). The combination of increased mtROS production and decreased ROS scavenging would potently drive oxidative stress and ROS-dependent cell death by mechanisms that might include ferroptosis (42).

Along with downregulation of TCA and ETC components, we observed SIRT3-dependent upregulation of *Hk2* and *Glut1* and increased lactate secretion following infection, indicating a shift toward glycolysis. These changes confirm and extend prior studies (3–5) and add to our earlier report of increased mitochondria-associated HK2 protein levels in M. tuberculosis-infected macrophages and a requirement of HK2 for ROS production (27). However, a recent study reported deceleration of both OXPHOS and glycolysis in human macrophages following M. tuberculosis infection (8). These contrasting results may be explained by differences in cell type, as well as different conditions of infection that bear further investigation.

We highlight SIRT3 in metabolic reprograming of M. tuberculosis-infected macrophages since it is the major mitochondrial sirtuin and is known to regulate mitochondrial and redox homeostasis (14, 16, 17, 54). However, the other mitochondrial sirtuins (SIRT4 and SIRT5) may also be involved. We found lower SIRT5 gene expression in J2 and WT macrophages after infection, while Yang et al. (55) reported that SIRT5 uniquely binds the complex I subunit protein NDUFV3. This suggest that SIRT5 could participate in regulating complex I activity following M. tuberculosis infection. While SIRT4 participates in resolving lipopolysaccharide-induced inflammation by regulating mitochondrial pyruvate dehydrogenase kinase I to rebalance mitochondrial respiration (56), we did not detect altered SIRT4 transcript levels. Further studies will be required to delineate the possible roles of SIRT4 and SIRT5 in M. tuberculosis-induced metabolic reprogramming in macrophages.

Taken together, our study provides evidence that downregulation of SIRT3 is important in the metabolic perturbation that occurs in macrophages following M. tuberculosis infection and that this impacts TB pathogenesis. Whether metabolic reprogramming benefits the host or pathogen is a complex question; both outcomes might be possible at different phases of the host-pathogen interaction. We speculate that the perturbation of mitochondrial homeostasis rapidly enhances innate host defense by increasing aerobic glycolysis and decreasing TCA cycle and ETC activity, but at the cost of mtROS accumulation. This may be followed by mitochondrial stress and macrophage necrosis that subsequently lead to a pathogen permissive inflammatory environment during chronic phase of infection.

## MATERIALS AND METHODS

Detailed methods are presented in the supplemental material (see Text S1 in particular).

10.1128/mBio.03140-20.7 TEXT S1 Supplemental materials and methods. Detailed descriptions of methods for M. tuberculosis infection and histopathology, quantitative real-time PCR, immunoblotting and immunoprecipitation, assays for ROS, metabolites, ETC enzymatic activity, cell necrosis, and cell viability, and SIRT3 overexpression are provided. Download Text S1, PDF file, 0.1 MB. Copyright © 2021 Smulan et al. 2021 Smulan et al. This content is distributed under the terms of the Creative Commons Attribution 4.0 International license.

### Mice.

Age-matched male C57BL/6J WT (JAX 000664) and *Sirt3^−/−^* [B6.129S6(Cg)-Sirt3^tm1.1Fwa^/J, JAX 027975] mice (17) were obtained from The Jackson Laboratory (Bar Harbor, ME). *Lyz2* (B6.129P2-Lyz2^tm1(cre)lfo^/J, JAX 004781) and *Sirt3^L2/L2^* mice [B6.129(Cg)-*Sirt3^tm1.1Auw^*/J, JAX 031201], obtained from The Jackson Laboratory (Bar Harbor, Me) (57, 58), were crossed to create *LysM^cre^Sirz^L2/L2^* mice. Male NADPH oxidase *gp91^phox–/–^* mice were obtained as described previously (59). *Myd88^−/−^/Trif^−/−^* mice were donated by K. Fitzgerald (University of Massachusetts Medical School [UMMS]). Mice were housed in the Animal Medicine facility at UMMS, where experiments were performed under a protocol approved by the Institutional Animal Care and Use Committee and Institutional Biosafety Committee.

### Cell isolation and culture.

Murine J2-immortalized macrophages from (WT) C57BL/6J mice, *Tlr2^−/−^* and *Tlr4^−/−^* mice, developed as described previously (60), were cultured in Dulbecco modified Eagle medium (DMEM) complete medium containing 10% fetal bovine serum, 0.1% 2-mercaptoethanol, and 2 mM l-glutamine. For RNA silencing, 24 h before infection, J2 macrophages were transfected with 20 pmol of siRNA targeting *Sirt3* (Thermo Fisher Scientific) or nontargeting control siRNA using Lipofectamine RNAiMAX (Thermo Fisher Scientific). For BMDM, bone marrow cells from 8- to 10-week-old age-matched male C57BL/6J, *Sirt3^−/−^*, *gp91^phox–/–^*, or *Myd88^−/−^/Trif^−/−^* mice were differentiated for 7 days in complete DMEM supplemented with 20% L929 supernatant. For all experiments, macrophages were split using 0.05% trypsin-EDTA, seeded at 6 × 10^5^ cells/ml, and incubated at 37°C with 5% CO_2_ for 24 h before infection. RPM were obtained by washing the peritoneum with complete DMEM and isolating CD11b^+^ cells using magnetic bead separation (Miltenyi Biotec). CD11b^+^ cells were seeded at 1 × 10^6^ cells/ml and maintained at 37°C with 5% CO_2_ for 24 h before infection.

### Statistical analysis.

GraphPad Prism 7.0 was used for statistical analysis. The results of experiments show combined data from three to four individual experiments and are expressed as the means ± the standard deviations (SD). For comparison to uninfected control cells, Student *t* test was used to analyze significance. One-way analysis of variance (ANOVA) was used when comparing three or more groups, and a log-rank (Mantel-Cox) test was used for the survival study. A *P* value of <0.05 was considered statistically significant.

10.1128/mBio.03140-20.6 TABLE S1 Primer sequences for real-time PCR. Primer sequences for *Sirt1* to *Sirt7*, *Idh1*, *Idh2*, *Nduf2*, and *Nduf7* are indicated. Download Table S1, PDF file, 0.1 MB. Copyright © 2021 Smulan et al. 2021 Smulan et al. This content is distributed under the terms of the Creative Commons Attribution 4.0 International license.
