# Supplementary figures and images for: Sirtuin 3 Downregulation in Mycobacterium tuberculosis-Infected Macrophages Reprograms Mitochondrial Metabolism and Promotes Cell Death
Source: mBio. 2021 Feb 2;12(1):e03140-20. doi: 10.1128/mBio.03140-20 (PMC7858060; doi:10.1128/mBio.03140-20)

Figure S1

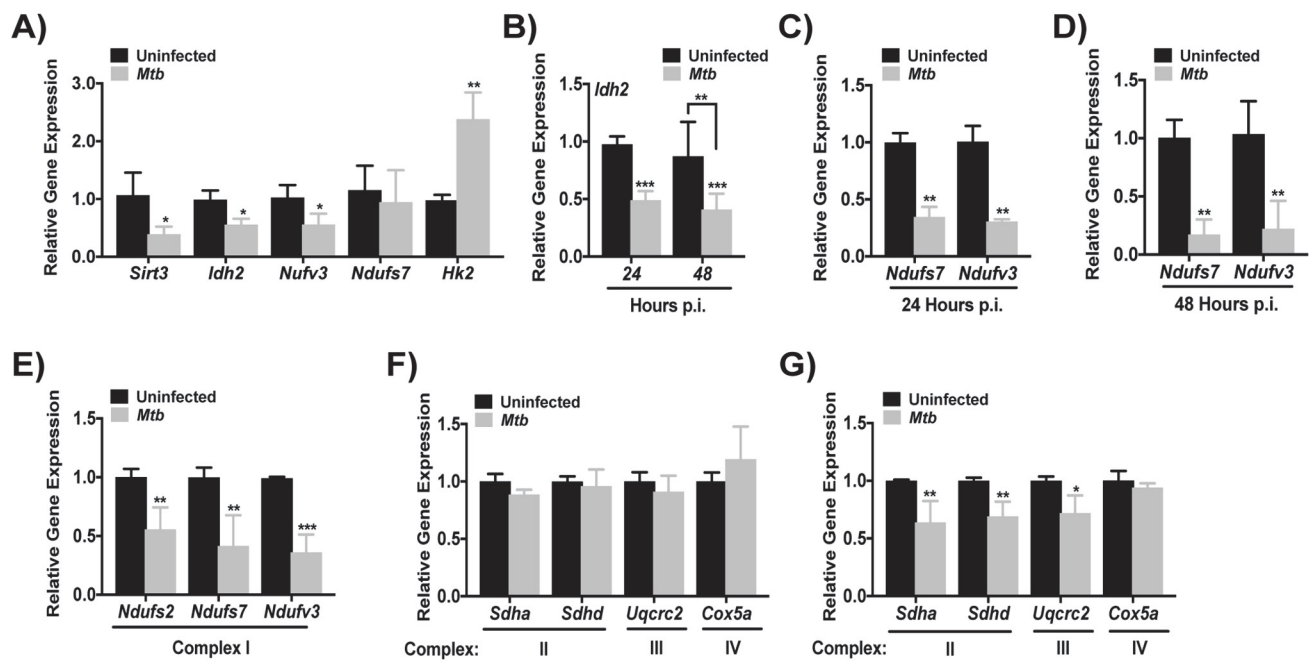

Supplement: FIG S1 [file mBio.03140-20-sf001.pdf]

Figure S2

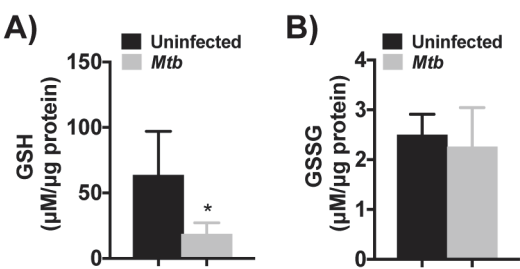

Supplement: FIG S2 [file mBio.03140-20-sf002.pdf]

Figure S3

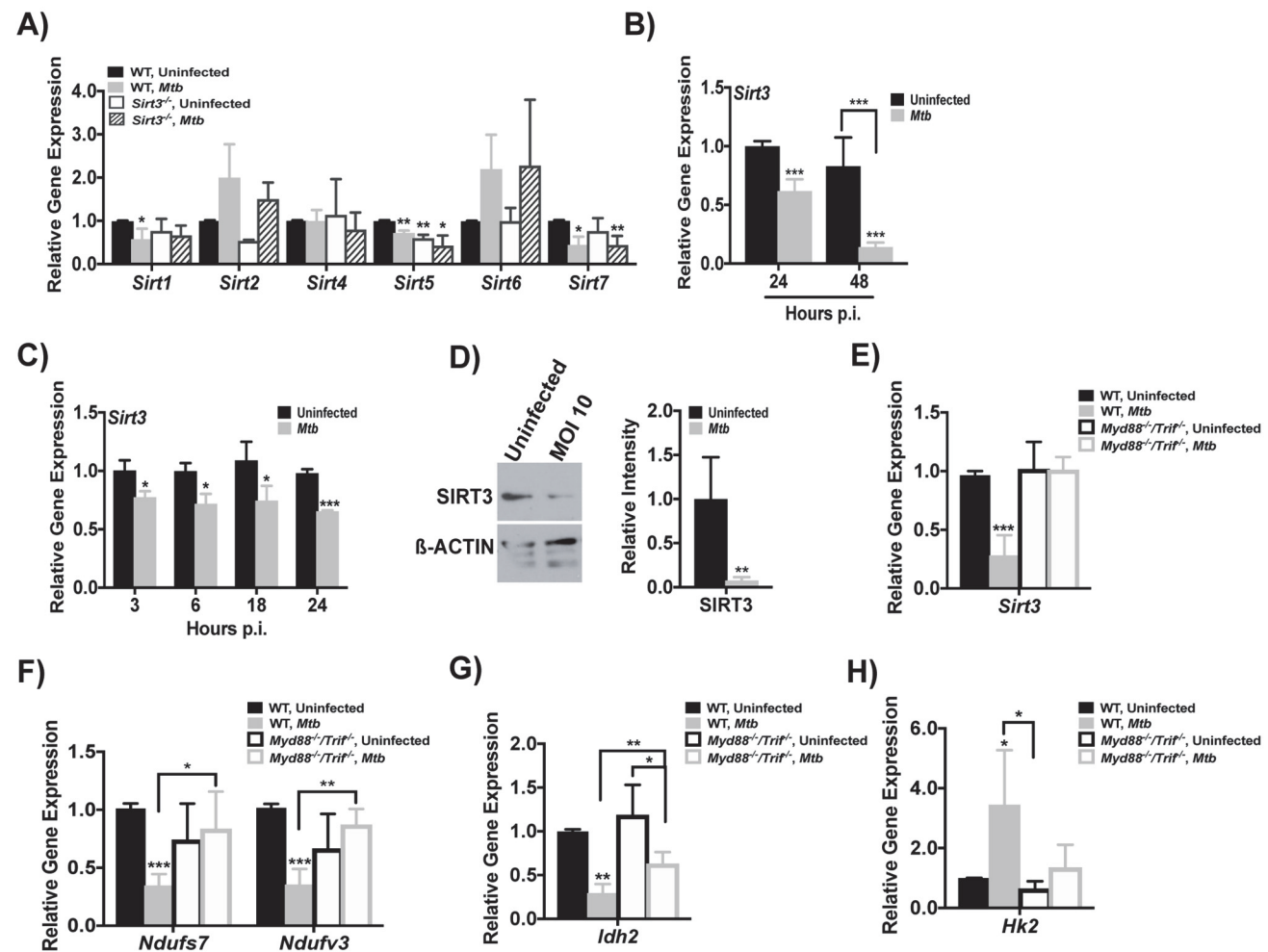

Supplement: FIG S3 [file mBio.03140-20-sf003.pdf]

Figure S4

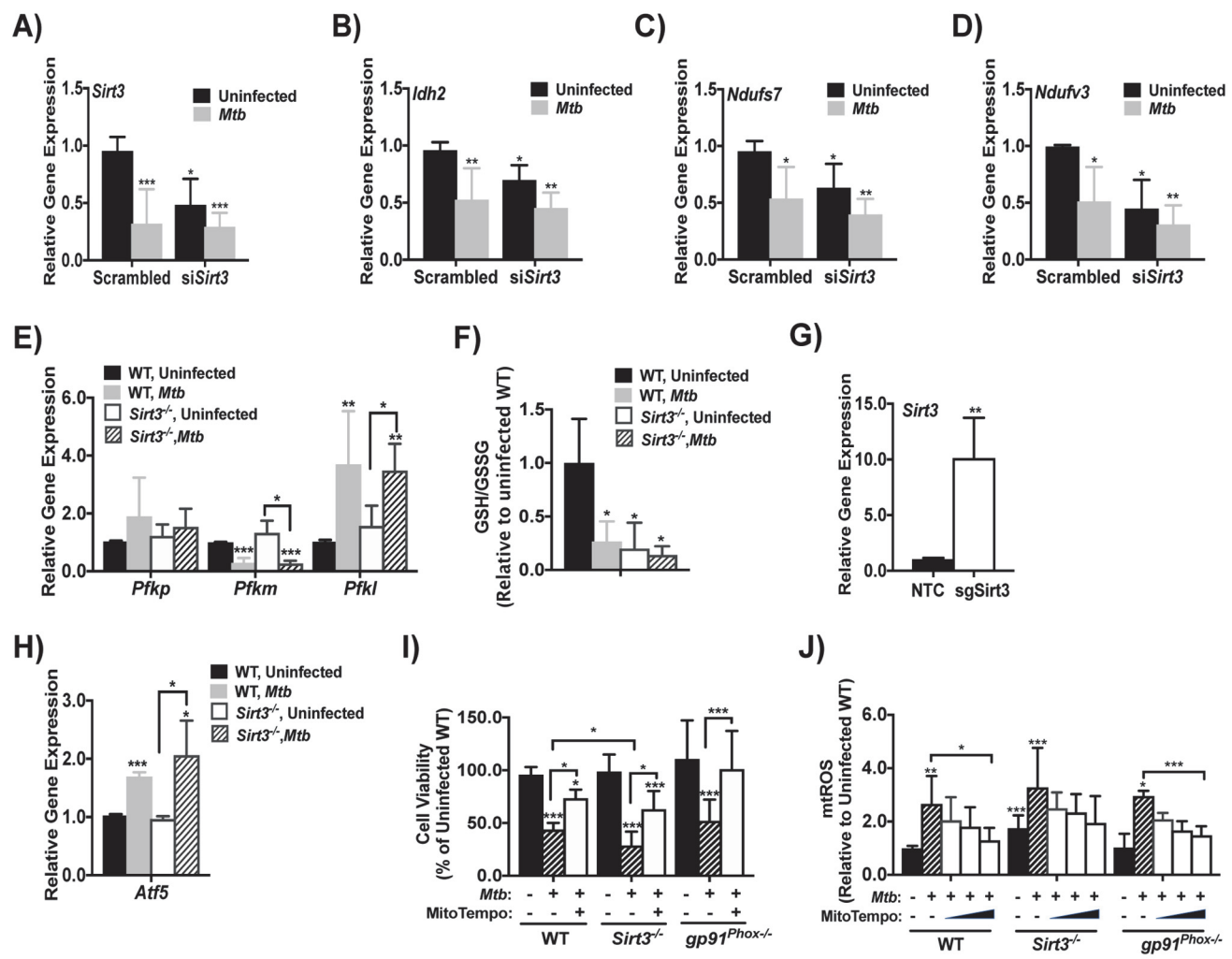

Supplement: FIG S4 [file mBio.03140-20-sf004.pdf]

Figure S5

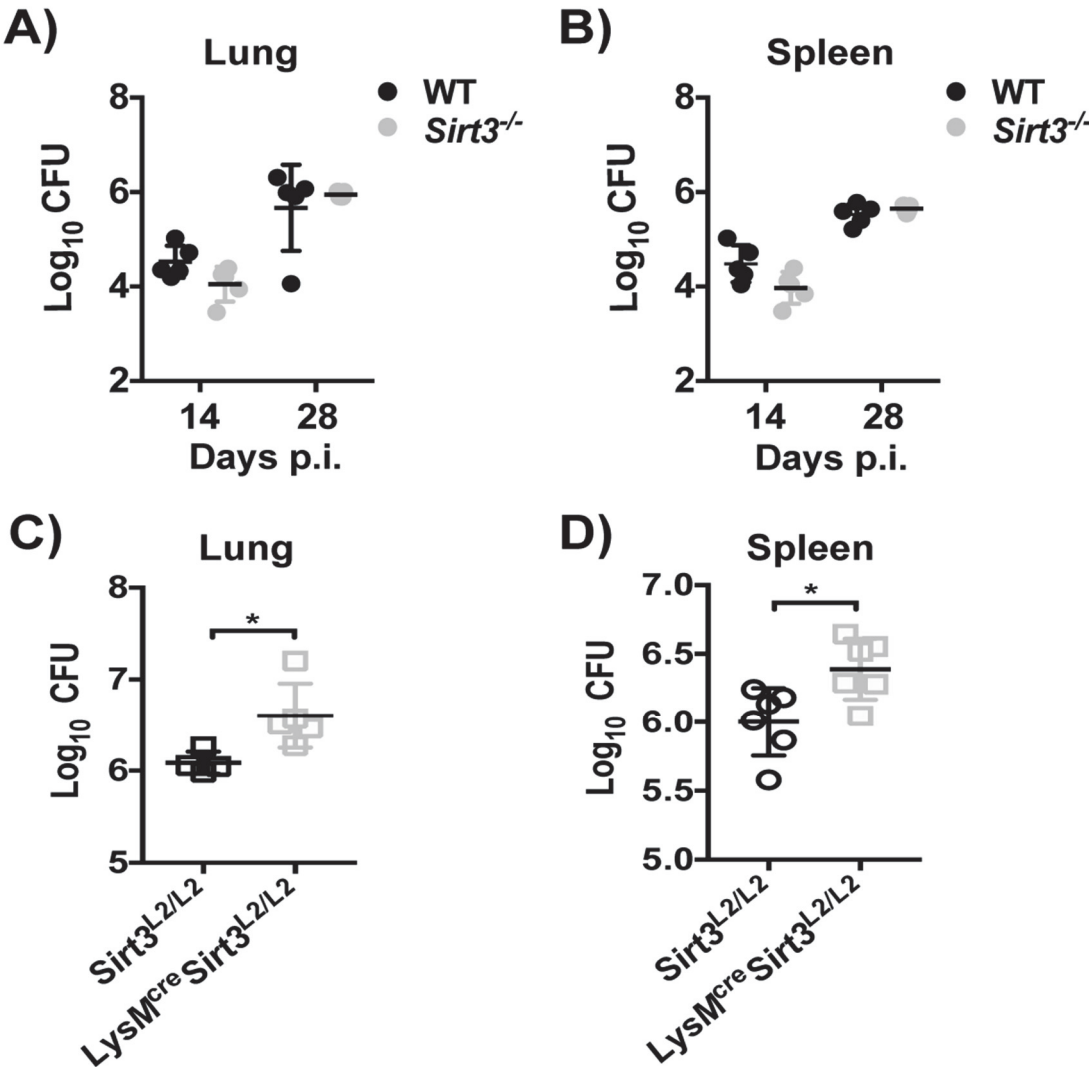

Supplement: FIG S5 [file mBio.03140-20-sf005.pdf]
