# Supplementary material for: Sirtuin 3 Downregulation in Mycobacterium tuberculosis-Infected Macrophages Reprograms Mitochondrial Metabolism and Promotes Cell Death
Source: mBio. 2021 Feb 2;12(1):e03140-20. doi: 10.1128/mBio.03140-20 (PMC7858060; doi:10.1128/mBio.03140-20)
Supplement: TEXT S1 [file mBio.03140-20-s0001.pdf]

Supplemental Materials and Methods

In vivo M. tuberculosis infection

Eight-week old male C57BL/6J or *Sirt3*$^{-/-}$ mice and male or female *LyzM*^{Cre}*Sirt3*$^{fl/fl}$ or *Sirt3*$^{fl/fl}$ littermate control mice were infected with *M. tuberculosis* Erdman by aerosol targeting ~100 CFU as described (1). The health of the mice was monitored weekly by body weight. Lungs and spleen were harvested from C57BL/6J or *Sirt3*$^{-/-}$ mice before death or at 2, 4, or 20 weeks p.i. and from *LyzM*^{Cre}*Sirt3*$^{fl/fl}$ or *Sirt3*$^{fl/fl}$ mice at 8 weeks p.i. for CFU determination and histopathology. For CD11b^{+} lung leukocyte isolation, infected C57BL/6J or *Sirt3*$^{-/-}$ mice were euthanized 16-weeks p.i. and lungs were minced in DMEM containing 10% fetal bovine serum. Single cell suspensions were obtained by adding 250 U/ml collagenase type IV in media, homogenized using a gentleMACS dissociator (Miltenyi Biotec) and then strained and treated with red blood cell lysis prior to CD11b^{+} cell isolation using magnetic bead separation (Miltenyi Biotec). The cells were seeded at 1 x 10^6 cells/mL before RNA and ROS analysis.

In vitro M. tuberculosis infection

For infection with *M. tuberculosis* Erdman, an aliquot of stock was sonicated for one minute prior to macrophage challenge at MOI 10. For infection with H37Rv or H37RVΔ*phoPR*, bacteria were grown in Middlebrook 7H9 broth supplemented with Middlebrook OADC enrichment to OD600 of 0.6-0.8, filtered (5.0µm), washed twice with PBS and resuspended in culture media prior to macrophage challenge at MOI 10. Infected macrophages were incubated (37°C, 5% CO_2) for 24 or 48 hours. Culture media from uninfected and infected cells was stored at -80°C. For selected experiments, macrophages were pre-treated with MitoTEMPO (50-500µM; Sigma-Aldrich),

Honokiol (10 or 20 µM; Cayman), or vehicle control for 3 hours before infection. MitoTEMPO and Honokiol were maintained for the duration of infection.

SIRT3 overexpression

For SIRT3 overexpression, J2 macrophages were transduced with a lentivirus containing catalytically inactive Cas9 fused to transcriptional activators (Addgene #61425) and selected with blasticidin at 5 µg/ml. Clones were screened for transcriptional induction following transduction with a test single guide RNA (sgRNA-Cd4). To generate a *Sirt3* overexpressing cell line, the clonal CRISPRa cell line was transduced with a lentivirus containing sgRNA specific for *Sirt3* (5'-GTAAGAGTGCCGAGAGGGCG-3') or a non-targeting control (5'-GCTTTCACGGAGGTTCGACG-3'), which were cloned into pXPR_502 (Addgene, #96923) by Golden Gate cloning. Cells were selected with 2.5 µg/ml puromycin. Lentivirus was generated using HEK293T transfection with TransIT-293 (MirusBio); HEK293T cells were transfected at a mas ratio of 6:4:2 (transgene : packaging (psPax2) : envelope (pVSV-G)). Viral supernatants were pooled 48 and 72 hours after transfection and clarified by centrifugation and filtration. Transductions were performed using polybrene (Santa Cruz Biotechnology) at 8 µg/ml.

Histopathology

Lungs were inflated and fixed in 10% buffered formalin for ≥24 hours, then processed for staining with hematoxylin and eosin (H&E) by the Morphology Core Facility at UMASS Medical School. Lung lesion and total lung areas were measured using ImageJ (2) and lesion area was expressed in relation to total lung area.

CFU determination

Spleens and lungs were homogenized in PBS-0.05% Tween 80, serially diluted and plated in duplicate on Middlebrook 7H11 agar plates supplemented with Middlebrook OADC enrichment. Plates were cultured at 37°C for four weeks prior to counting colonies.

Gene Expression

RNA isolated using Quick-RNA MicroPrep Kit (Zymo Research) was reverse-transcribed using the High Capacity Reverse Transcription kit (Applied Biosystems. Real-time PCR was performed using AzuraQuant Green Fast qPCR Mix LoROX and a CFX96 Real-Time System (Bio-Rad). Primer sequences are listed in Table S1. Data normalized to β-ACTIN (*Actb*) and TATA-box binding protein (*Tbp*) and calculated by delta-delta Ct method are expressed as fold change compared to uninfected cells.

Immunoblotting and immunoprecipitation

J2 macrophages or BMDM were lysed in RIPA buffer (50 mM Tris, pH 8.0, 150 mM NaCl, 0.5% sodium deoxycholate, 1.0% Triton X-100, 0.1% sodium dodecyl sulfate [SDS]) containing proteinase inhibitors. Protein was measured by BCA assay and 20 µg of sample was separated by SDS-polyacrylamide gel electrophoresis, then transferred onto Immun-Blot PVDF membranes (Bio-Rad). Membranes were blocked in 5% milk and incubated with primary antibody overnight at 4°C. Primary antibodies included: SirT3 (D22A3) Rabbit (Cell Signaling Technology), IDH1 (D2H1) Rabbit (Cell Signaling Technology), IDH2 (D8E3B) Rabbit (Cell Signaling Technology), ß-Actin (13E5) Rabbit (Cell Signaling Technology). Following incubation with

horseradish peroxidase-conjugated secondary antibodies, chemiluminescence reagent was added to the membranes and exposed to Hyblot CL Autoradiography film (Denville Scientific). Protein bands were quantified using Image (2). For immunoprecipitation, 1mg of J2 macrophage cell lysates were incubated with Acetylated-Lysine antibody (Cell Signaling Technology) overnight, followed with Protein A Dynabeads (ThermoFisher Scientific) at 4°C for 2 h. Complexes were washed five times with lysis buffer, then eluted from beads with elution buffer (50mM Glycine, pH 2.8) and SDS sample buffer. Samples were heated at 70°C. Immunoblot analysis was performing as above.

Reactive oxygen species assay

Macrophages were seeded in 96-well plates and analyzed for cellular and mtROS 24 hours p.i. Cellular ROS was measured using the DCFDA cellular ROS detection Kit (Abcam). For mtROS analysis, macrophages were incubated in PBS containing 5 µM MitoSOX Red Mitochondrial Superoxide Indicator (ThermoFisher Scientific) at 37°C for 30 minutes. Emitted Fluorescence (Ex/Em = 510/580) was measured.

Necrosis and cell viability assay

Cells seeded in 96-well plates were treated with Ethidium Homodimer-1 (ThermoFisher Scientific) at a final concentration of 2 µM, infected and emitted fluorescence was measured 24 hours p.i. For the cell viability assay, macrophages were seeded in 96-well plates and infected for 24 hours with *M. tuberculosis*. 100 µl of CellTiter-Glo Luminescent Cell Viability Assay (Promega) was added to 100 µl of culture media, allowed to stabilize for 10 min, and luminescence was recorded using a Synergy H4 hybrid plate reader plate reader.

Metabolite analysis and Complex activity assay

Macrophages were washed twice with PBS, scraped in PBS and pelleted by centrifugation (300 x g, 5 minutes). Cell pellets were lysed in RIPA buffer, centrifuged to remove debris, and deproteinated using metaphosphoric acid. To increase the sample pH, triethanolamine was added before assaying for GSH and GSSG using the Glutathione Assay Kit (Cayman), and total isocitrate using the Isocitrate Assay Kit (Sigma-Aldrich). Secreted lactate was measured in media from uninfected and infected J2 macrophages and BMDM experiments using the Lactate Assay Kit (Sigma-Aldrich). Complex I and II enzymatic activity were assayed using the Complex I or Complex II Enzyme Activity Microplate Assay Kit (Abcam). Briefly, J2 macrophages were washed and pelleted as above. Pellets were resuspended in PBS to make a 5 mg/ml cell suspension for the assays per the manufacturer's instructions.

References

1. Repasy T, Lee J, Marino S, Martinez N, Kirschner DE, Hendricks G, Baker S, Wilson AA, Kotton DN, Kornfeld H. PLoS Pathog 9:e1003190, 2013, PMC3578792

2. Schneider CA, Rasband WS, Eliceiri KW. Nat Methods 9:671-5, 2012, PMC5554542
