# Supplementary material for: Sirtuin 3 Downregulation in Mycobacterium tuberculosis-Infected Macrophages Reprograms Mitochondrial Metabolism and Promotes Cell Death
Source: mBio. 2021 Feb 2;12(1):e03140-20. doi: 10.1128/mBio.03140-20 (PMC7858060; doi:10.1128/mBio.03140-20)
Supplement: TABLE S1 [file mBio.03140-20-st001.pdf]

Table S1. Primer Sequences for Real-time PCR

Gene Name	Accession Number	Forward Primer	Reverse Primer
Sirt1	NM_019812	GATGACAGAACGTCACACGC	ATTGTTCGAGGATCGGTGCC
Sirt2	NM_022432	CTCTGACCCTCTGGAGACCC	ATCTCTGCCTCTCCACCAGT
Sirt3	NM_022433.2	TGCCTGCAAGGTTCCTACTC	AGTCGGGGCACTGATTTCTG
Sirt4	NM_001167691.1	ATTCCCGCTGTGGAGAGTTG	TTCAGAGTTGGAGCGGCATT
Sirt5	NM_178848	CTTTTTGCAGCCTGCCTGG	GCAGGAGGCTTTCGTCTACA
Sirt6	NM_181586	AAGTCTCACTGTGTCCCTTGTC	TCACGAGCGGGTGTGATTG
Sirt7	NM_153056	TCTACAACCGGTGGCAGGAT	AGTGACTTCCTACTGTGGCTG
Idh1	NM_001111320	GAGACCATTGAGGCTGGCTT	GGCCTGAGCTAATTTGGCCT
Idh2	NM_173011.2	TTACCGAGAACACCAGAAGGG	TAAGGTCCTGGTTCCCATCC
Ndufs2	NM_153064	AACAGCGGAAGACGCACTTA	CCACCTGGTCGTAAACGTCA
Ndufs7	NM_029272	AGAGTTCATCAGAGTGTAGCCA	GAAGATGAGAGAGCTTGGGGAC
